# RESCUE - reduction of MR-SNR-degradation by using an MR-synchronous low interfering PET acquisition technique

**DOI:** 10.1186/2197-7364-1-S1-A87

**Published:** 2014-07-29

**Authors:** Pierre Gebhardt, Bjoern Weissler, Jakob Wehner, Thomas Frach, Paul Marsden, Volkmar Schulz

**Affiliations:** King’s College London, London, UK; RWTH Aachen University, Aachen, Germany; Philips Research Europe, Aachen, Germany; Philips Digital Photon Counting, Aachen, Germany

The combination of PET and MRI is a challenging task, since both imaging modalities might influence each other. For unaffected imaging performance, PET detectors need to be untouched by the MR operation (MR-compatible) and RF-silent. The latter requirement is demanding, as the digital Data Acquisition and Control Architecture (DACA) produce noise in the MRI receive chain and thus deteriorate the Signal-to-Noise Ratio (SNR). To reduce these MRI-SNR degradations, we propose “RESCUE”, a MR-synchronously gated PET data acquisition technique. By controlling the PET-modules’ digital data acquisition in terms of interrupting the digital SiPM (dSiPM) operation during the MR signal receive phases, potential RF interferences can be reduced. The concept and design of RESCUE are based on the DACA of Hyperion-II^D^ using 960 dSiPMs from Philips which are spread over 10 PET modules, every module containing 6 detector stacks. 16 dSiPMs are populated on one detector stack and a local FPGA per stack is used to clock and configure the dSiPMs and acquire data from them.

To interrupt the PET data acquisition when the MRI is in receive-mode, an MRI trigger signal is sent via the backbone of the PET insert to all PET modules. In the stacks, the dSiPM-operation is interrupted by gating the dSiPMs’ input clock signals. This clock switch-off needs to be performed at a point in time when no dSiPM cell recharge takes place, as sensor-damage might otherwise occur.

We implemented FPGA firmware to stop the above described dSiPM operation and to restart it upon release of the gating. PET-data was acquired using gating-sequences and analysis of sensor data acquired before and after interruption showed that the sensors were working properly after reapplying their clock signals.

More details and MRI SNR-measurement results to demonstrate the interference reduction obtained with RESCUE will be presented at the conference.

